# Landscape heterogeneity and pesticide reduction favor predation, but also grape infestation by *Lobesia botrana*

**DOI:** 10.1002/eap.70045

**Published:** 2025-06-03

**Authors:** Axelle Tortosa, Aude Vialatte, Fabien Laroche, Adrien Rusch, Martin H. Entling, Brice Giffard

**Affiliations:** ^1^ Université de Toulouse, INRAE, DYNAFOR Castanet‐Tolosan France; ^2^ INRAE, ISVV, UMR 1065, Santé et Agroécologie du Vignoble Villenave d'Ornon France; ^3^ iES Landau, Institute for Environmental Sciences, RPTU University of Kaiserslautern‐Landau Landau Germany; ^4^ Bordeaux Sciences Agro, INRAE, ISVV, SAVE Villenave d'Ornon France

**Keywords:** complementarity, farming practices, landscape, *Lobesia botrana*, natural pest control, *Vitis vinifera*

## Abstract

Biological pest control is a major ecosystem service and is known to depend on landscape heterogeneity. The composition and configuration of landscapes can affect natural enemy communities, trophic interactions, and pest density within agroecosystems. However, local agricultural management can interfere with natural enemy activity, so the positive effects of landscape heterogeneity may be disrupted by farming practices. Here, we studied the influence of landscape context and management options on the biological control of *Lobesia botrana*, one of the main insect pests of grapes. We focused on two complementary measures: predation rates, which reflect part of biological control potential, and plant damage, which reflects pest density and the associated infestation. We used a set of sentinel prey (eggs, caterpillars, pupae) to quantify predation rates across different developmental stages of the pest. The study was carried out in a landscape‐scale experimental set‐up consisting of 38 vineyards in Southwestern France. Using structural equation models, we show that predation rates on sentinel prey were affected by both landscape heterogeneity and local management practices. Higher pest predation rates were observed in landscapes with smaller vineyards and in vineyards with low applications of synthetic pesticides. We observed limited relationships between predation rates and grape infestation levels. However, our results suggest that predation rates at the pest pupae stage are significantly shaping infestation levels. Additionally, pest damage in spring and summer was primarily influenced by the intensity of local pesticide use and the grass cover in the field and exacerbated by the decreasing size of vineyards, while semi‐natural habitats had no effect on pest damage. We conclude that links between *L. botrana* infestation and biological control potential appear tenuous in our study region. This is likely due to the high local management intensity, as evidenced by the negative association observed between pesticide applications and predation rates. Nevertheless, both predation and infestation respond to landscape or field heterogeneity and pesticide use. Reducing the use of pesticides should be combined with multi‐scale diversification measures at field and landscape levels to amplify the predation potential.

## INTRODUCTION

Crop damage by pests is a major challenge for farmers given that pests are responsible for around 20% of yield losses on a global scale (Duflot et al., 2022; Oerke, 2006). Pest attacks are predicted to increase due to global change (Simler‐Williamson et al., 2019). For instance, rising temperatures can affect pest dynamics by increasing the number of generations per year and enhancing earlier emergence (Martín‐Vertedor et al., 2010). Additionally, elevated CO₂ levels may alter host–pest–pathogen interactions and further influence the geographical distribution of agricultural pests (Skendžić et al., 2021). At the same time, the intensive use of synthetic pesticides has led to major impacts on biodiversity and ecosystem services, including biological pest control (Billeter et al., 2008; Flynn et al., 2009; Geiger et al., 2010), which is a key service for crop production (Dainese et al., 2019). Therefore, reducing the dependence on synthetic pesticides for pest control is of major importance.

Numerous studies have demonstrated that landscape heterogeneity and crop diversity benefit natural enemies, which in turn limit pest damage (Beaumelle et al., 2021; Estrada‐Carmona et al., 2022; Letourneau et al., 2011; Perrot et al., 2023; Redlich et al., 2018). The natural enemies hypothesis predicts that heterogeneous landscapes, supporting more than 20% of semi‐natural habitats (Boetzl, Schuele, et al., 2020), promote more abundant or diverse natural enemy communities (Langellotto & Denno, 2004). Landscape spatiotemporal heterogeneity supplies not only complementary or supplementary resources, such as food sources, but also shelter, nesting, and overwintering sites that benefit natural enemies and ultimately pest control services (Bertrand et al., 2012; Clough et al., 2020; Garibaldi et al., 2018; Root, 1973). Moreover, landscape configuration, such as the average size of agricultural fields, affects natural enemy communities and their movements, as well as pest populations (Clough et al., 2020; Fahrig et al., 2015). Heterogeneous landscapes with smaller fields have high density of edges and a higher diversity of habitats, resulting in a higher potential for spill‐over of natural enemies (e.g., arthropods, birds, parasitoids) from non‐crop habitats (Fahrig, 2017; Martin et al., 2019; Smith et al., 2014).

However, pest control services delivered by natural enemies may not increase linearly with increasing landscape heterogeneity and, in some cases, may fail to increase at all (Karp et al., 2018). Highly heterogeneous landscapes may promote higher competition and/or intraguild predation between natural enemies, limiting biological pest control (Martin et al., 2013). Additionally, as noted by Tscharntke et al. (2016), natural habitats may sometimes fail to enhance pest control, for example, due to the absence of effective natural enemies, agricultural practices that disrupt enemy populations, or even natural habitats acting as sources of pests. Conversely, a positive relationship between landscape heterogeneity and pest control does not necessarily indicate a top‐down effect from natural enemies. Lower pest abundances in more heterogeneous landscapes can also emerge due to direct effects of the landscape on the pest population dynamics. For example, heterogeneous landscapes with higher plant diversity can induce chemical and/or physical host disruption (Andow, 1991; Chaplin‐Kramer et al., 2011) leading to a lower probability for pests finding their host plants and then feeding or reproducing, that is, the resource concentration hypothesis (Root, 1973). How these two non‐exclusive hypotheses, that is, the natural enemy and the resource concentration hypothesis, actually shape pest control services in agricultural landscapes remains understudied, with few studies investigating how these mechanisms interact to influence pest control (e.g., Plata et al., 2024).

It is now widely admitted that farming practices may counteract the benefits of landscape heterogeneity by affecting natural enemies communities (Ricci et al., 2019; Winqvist et al., 2011). On the contrary, the presence of semi‐natural habitats can foster positive effects of less‐intensive farming practices such as organic farming on natural enemies communities and biological pest control (Klinnert et al., 2024; Muneret et al., 2019; Winqvist et al., 2011). However, these effects remain poorly understood, as they are taxa‐dependent (Muneret et al., 2018; Ostandie, Muneret, et al., 2021) and because there is a large variability in the intensity of practices, even within organic fields (Gosme et al., 2012; Puech et al., 2014). The differences between organic and conventional systems are not always consistent, especially in perennial crops such as vineyards. For instance, the management of organic vineyards can rely on large amounts of non‐synthetic pesticides (e.g., fungicides with copper and sulfur) with negative effects on biodiversity (Karimi et al., 2021; Reiff et al., 2023). Moreover, the management of inter‐row vegetation in vineyards does not necessarily differ between organic and conventional fields, whereas intensive tillage has been shown to reduce biodiversity (Giffard et al., 2022; Paiola et al., 2020; Winter et al., 2018). Finally, although the use of pesticides intends to reduce pest densities within fields, it can have unintended impacts on the resources and dynamics of natural enemies (Janssen & van Rijn, 2021). Because landscape context and farming practices directly or indirectly affect pest populations, it is important to disentangle the relative effects of host density (i.e., plants targeted by pests), landscape diversity, and farming practices on the level of biological pest control (Tscharntke et al., 2016). This remains poorly understood, especially in perennial systems, such as in vineyards.

The European grapevine moth *Lobesia botrana* (Lepidoptera, Tortricidae) is among the most damaging insects in European vineyards (Delbac & Thiéry, 2016; Moschos, 2006; Thiéry, 2008). Yield losses vary not only between years but also among the different generations occurring during the growing season. In the southwest of France, grape berry moths often develop a third generation before the harvest, depreciating not only grape quantity but also quality and causing higher economic losses than the first two generations. Biological pest control is an ecologically and economically promising solution but represents a major challenge as vineyards are one of the crops with the most intensive use of pesticides (Fouillet et al., 2022). All developmental stages of *L. botrana*—egg, larvae, pupae, and moth—are predated by several natural enemies, such as arthropod predators or parasitoids, avian, and mammalian predators (Rusch et al., 2017; Thiéry et al., 2018). Previous studies have shown the potential complementarity between natural enemy guilds, especially between parasitoids and predators in both annual and perennial agricultural systems, and the expected positive relationship between functional diversity of natural enemies and biological pest control (e.g., Dainese et al., 2017). Parasitoids can play a critical role in the biological control of *L. botrana*, particularly at larval stages, contributing significantly to pest regulation (Xuéreb & Thiéry, 2006). However, *L. botrana* exhibits a high level of plasticity in its response to parasitoids, including the ability to accelerate larval development under high parasitism pressure, which can reduce the effectiveness of this control strategy (Vogelweith et al., 2013). Our study focuses on the complementarity of predation across different life stages of *L. botrana*, without including parasitoids. The exact roles of natural enemy functional groups and their complementary effects on different pest stages are still not fully understood. Higher levels of biological pest control may result from niche complementarity between different enemies. For instance, carabid beetles may be involved in pupae predation (Thiéry et al., 2018), while birds and arthropods such as harvestmen can ensure larvae predation (Barbaro et al., 2017; Papura et al., 2020). Flying adult moths are mainly predated by bats (Charbonnier et al., 2021), while ants, spiders, or earwigs are involved in egg predation (Ostandie, Giffard, et al., 2021; Ostandie, Muneret, et al., 2021; Thiéry et al., 2018). To our knowledge, no study has explored the complementarity of predation across different life stages of grape berry moths. The presence of different functional groups of enemies can also lead to synergistic predation (Sih et al., 1998), resulting in higher predation rates at several stages or at a particular stage.

In this study, we investigated how landscape heterogeneity and farming practices affect predation of *L. botrana* at different developmental stages and the direct and indirect effects on crop damage in French vineyards. Thus, we evaluated the potential synergies between predation at different stages and the result in biological pest control efficiency in spring and summer during the first two generations of *L. botrana*. Through the construction of structural equation models (SEMs), we tested two alternative hypotheses: (H1a) pest infestation levels are determined by predation rates by natural enemies across different stages, resulting in lower pest density with higher predation rates over time, that is, top‐down processes (Mäntylä et al., 2011; Martin et al., 2013); or (H1b) the predator community and the level of predation rates are driven by pest abundance, with higher predation rates at higher pest infestation levels, that is, density‐dependent bottom‐up processes (Almdal & Costamagna, 2023). In addition, we predicted that (H2) landscape heterogeneity in the form of a higher amount of semi‐natural habitats and smaller vineyards favors higher levels of predation, lower pest densities, and higher complementarity across time resulting in reduced crop damage (Sirami et al., 2019; Tscharntke et al., 2021). We also expected (H3) consistent effects of biodiversity‐friendly practices in organic fields and in landscapes dominated by organic farming: we predicted higher predation rates in less‐intensively managed vineyards located in organic vineyard‐dominated landscapes (Beaumelle et al., 2023; Brusse et al., 2024). We expected (H4) that landscape complexity would influence farming practices, which would in turn indirectly affect biological control (Etienne et al., 2022; Ricci et al., 2019). Finally, (H5) while the distribution of predation pressure across pest developmental stages is uncertain, we predicted that combining predation at different developmental stages could enhance biological pest control. This was built on evidence of niche complementarity among natural enemies, which may lead to synergistic effects on pest suppression (Dainese et al., 2017; Straub & Snyder, 2008).

## METHODS

### Study area and landscape metrics

The study area was located in the southwest of France (44°48′ N, 0°14′ W) in a vineyard‐dominated region (Ostandie, Giffard, et al., 2021). Within this study area, 19 pairs of vineyards were selected along two landscape gradients. Each pair contained one organic vineyard and one non‐organic vineyard; in total, 38 fields were recorded. The mean patch size of vineyards ranged from 4742 to 11,732 m^2^. Within each landscape pair, the organic and conventional vineyards were either adjacent or slightly distant, with a maximum distance of 430 m between their centers (for vineyard locations, details are provided in supporting information of Beaumelle et al., 2023). These pairs were chosen along two landscape gradients estimated in a 500‐m radius and a 1‐km radius: a gradient of semi‐natural habitats (ranging from 1.7% to 62.6% in a 500‐m radius and 1.5% to 78.4% in a 1‐km radius) and a gradient of the proportion of organic vineyards (ranging from 2% to 58.1% in a 500‐m radius and 1.2% to 28.1% in a 1‐km radius). These commonly used scales were selected to capture complementary ecological effects: the 500‐m radius represents more localized influences of landscape metrics on vineyard biodiversity such as spiders and other arthropods and results in terms of biological pest control, while the 1‐km radius reflects broader landscape dynamics, including more mobile natural enemies like birds and bats (e.g., Chávez et al., 2025; Le Provost et al., 2021; Moraga et al., 2019; Redlich et al., 2018). These two gradients were slightly negatively correlated, but this correlation was not significant (Pearson's *r* = −0.34 and −0.55, respectively, at 500‐m and 1‐km radii). We considered several landscape metrics, which represented both landscape composition and configuration, to evaluate their direct effects on predation rates and their direct and indirect effects on crop damage. To examine the effects of landscape composition, we used the proportion of total semi‐natural habitats (i.e., forests, hedgerows, heathlands, permanent and temporary grasslands), woody semi‐natural habitats (i.e., forests and hedgerows), vineyards, and the proportion of certified organic vineyards. Landscape configuration was characterized by the mean patch size of vineyards. As we know that the influence of the landscape on predator communities and biological control can depend on the spatial scale considered, we calculated these landscape metrics in two buffers of 500‐m and 1‐km radii around the center of the field (Appendix S1: Figure S1). We obtained the land‐cover map of the study area by combining GIS layers from the Soil Occupancy Product OSO 2019 (Inglada et al., 2017), the Land Parcel Information System for organic farming (*Registre Parcellaire Graphique* [RPG], 2017), and field surveys to complete missing data on organic vineyards. We used QGIS 3.10 software and the sf package in R (Pebesma, 2018; Pebesma & Bivand, 2023) to calculate all landscape metrics.

### Local management

Wine growers were interviewed between November 2019 and February 2020 to collect data on farming practices used within vineyards during the growing period in 2019. The treatment frequency index (TFI) estimates the intensity of pesticide use and especially summarizes the overall dependence on these products (Lechenet et al., 2014). It is calculated for each pesticide application as the ratio between the applied dose and the recommended dose (regulatory dose that is efficient against pests and limits potential ecotoxicological effects on organisms other than pests), weighted by the treated surface area relative to the total plot surface area. Specifically, the TFI for a single application is given by the formula (Equation 1):

(1) $$\mathrm{TFI}=\frac{\text{Application rate}\times \text{Treated surface area}}{\text{Recommended dose}\times \text{Plot surface area}\;}.$$

This calculation is performed separately for each type of pesticide (herbicides, fungicides, and insecticides). The total TFI for each category is then obtained by summing the TFI values of all individual applications within the crop season. Organic vineyards only used copper‐ and sulfur‐based fungicides, while conventional vineyards used a range of synthetic and non‐synthetic substances to control fungal pathogens. Insecticides are used in both conventional and organic fields against tortricid moths (*L. botrana* and *Eupoecilia ambiguella*) and leafhoppers (Cicadellidae) with mandatory treatments to reduce the risk associated with the “flavescence dorée” disease, transmitted by *Scaphoideus titanus* (Ball). Herbicides are only used within rows and in conventional vineyards. We also used the copper quantity applied (in kilograms per hectare) to characterize the level of use of non‐synthetic pesticides in organic fields especially. The proportion of ground vegetation within fields (herbaceous cover in inter‐rows) was also calculated in all vineyards.

Principal components analysis (PCA) of farming practices (Appendix S1: Figure S2) revealed the differences between organic and conventional systems, mainly driven by total amounts of applied synthetic fungicides, insecticides, herbicides, and, at the opposite, some variation of the quantity of copper‐based fungicides used in organic fields.

### Pest predation

We used semi‐experimental sentinel approaches to quantify predation rates on pupae, egg, and caterpillar stages of *L. botrana*. First, we assessed predation on *L. botrana* eggs and pupae using sentinel cards. The cards were composed of five pupae placed on a 10 × 4 cm reinforced tape on which sand was added to avoid trapping the predators. At the top of the cards, a strip with 20–100 laboratory‐reared eggs of *L. botrana* was placed. In each vineyard, five sentinel cards were placed 5 m from each other in the central row, between 5 and 25 m from the edge. Each card was attached to the two‐year branch of the vine. After a 3‐day exposure, the cards were collected and the numbers of remaining pupae and eggs were counted, using a magnifying binocular in the laboratory for eggs (Muneret et al., 2019). We then estimated predation rates for each card and development stage as the ratio of the number of prey predated to the total number initially exposed. Predation measures on sentinel prey cards took place twice, at the beginning of June 2019 and the end of July 2019 (Appendix S1: Figure S3), which corresponded to the first and second generations of *L. botrana*, respectively.

We assessed caterpillar predation using plasticine dummy caterpillars mimicking pest larvae. Each larva was made of white, inodorous plasticine (Plastiline ivoire—Hardness 1, Cultura, France) and shaped to reasonably mimic the grape berry moth larvae (Barbaro et al., 2017; Beaumelle et al., 2023; Ostandie et al., 2022). In each vineyard, 30 plasticine caterpillars were fixed on 10 vine stocks. Five vine stocks were located at vineyard edges (5 m from the edge), and five vine stocks were located 25 m from the edge. Plasticine caterpillars were exposed for 13–15 days (between 21 May and 7 June 2019), and we recorded predation marks by a range of predators such as arthropods, birds, and bats (Barbaro et al., 2017). Predation rates at the caterpillar stage were then estimated as the proportion of dummy caterpillars showing marks of predator attack left at the end of the experimental period per plant (Barbaro et al., 2014).

### Grape berry moth damage

In each vineyard, we measured the damage caused by first (end of May to early June) and second generations (end of July) of the grape berry moth larvae. First‐generation larvae formed silk nests by agglomerating flower buds with silk threads. These nests represent damage, as flower buds are consumed, although such losses are often negligible due to compensation by larger berries. Each nest generally corresponds to one or two larvae (Delbac & Thiéry, 2017), making it a proxy for larval density and pest pressure for subsequent generations. Silk nests were monitored in 4–5 different vine rows and along a transect of 100 randomly selected and independent grape clusters in different vine stock, at the end of May, avoiding the edge of the fields.

Second‐generation larvae can cause greater damage and lower grape productivity, as they attack developing grapes, perforating them. These perforations are also a proxy for larval density, as one perforation generally corresponds to one larva.

### Statistical analysis: SEM

We used structural equation modeling (Rosseel, 2012) to disentangle (1) whether predation activity is negatively or positively correlated with pest density, reflecting either effective pest control driven by predator communities (H1a) or pest density dependence (H1b). We further examined (2) how landscape metrics and farming practices influence directly and indirectly predation types and levels of infestations (H2, H3, and H4) and (3) how the predation types influence infestation (H5).

**FIGURE 1:**
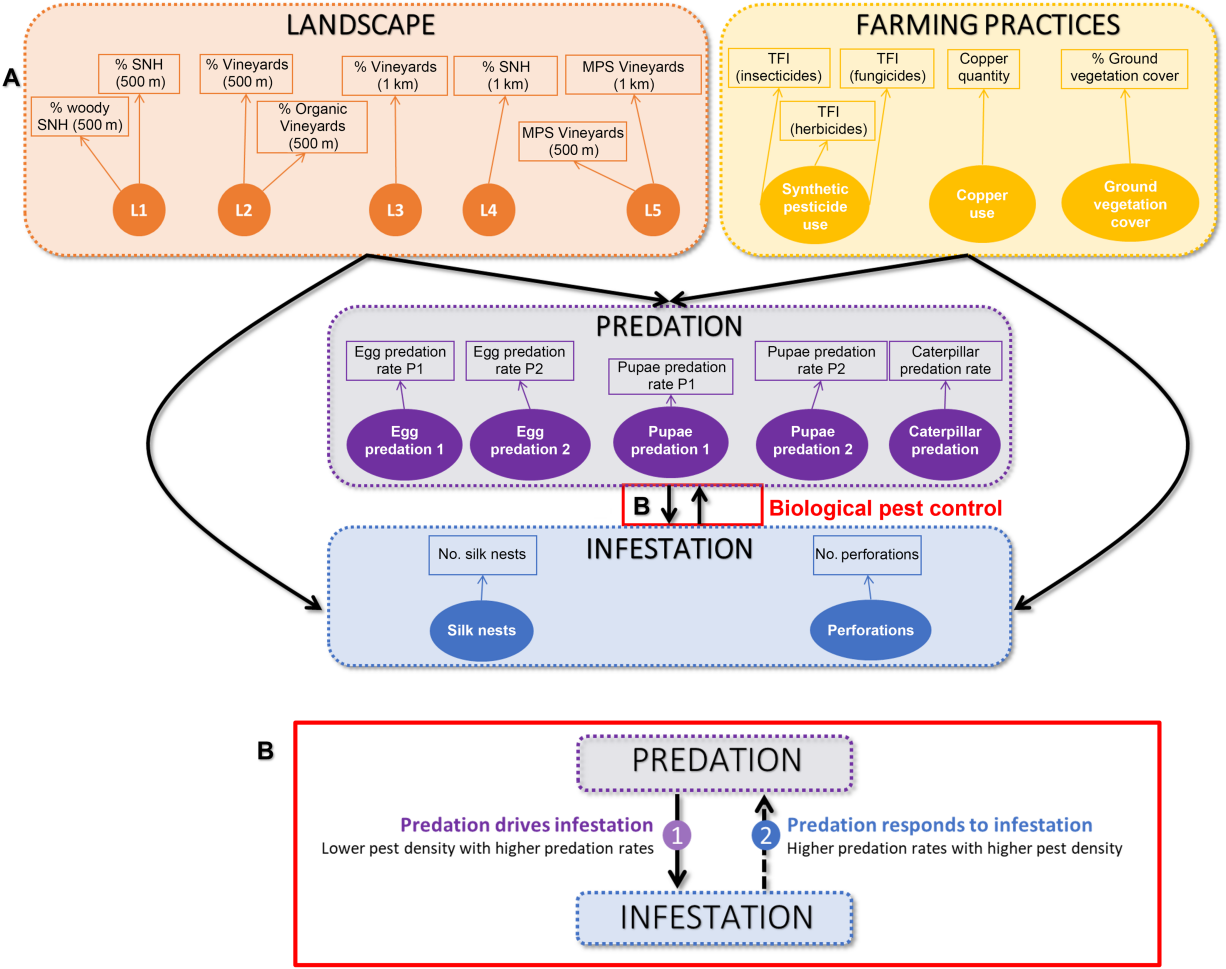
Structural equation model (SEM) depicting relationships between landscape context, farming practices, predation rates, and pest damage. (A) Conceptual framework of the SEM model. Circles represent latent variables, and boxes represent observed variables that serve as indicators of the latent variables. Latent variables relating to the same function or describing a same factor are grouped together graphically only in dotted boxes of the same color. Black arrows represent the effects of each latent variable within the boxes on each latent variable in the target box. Correlations between latent variables are not represented in this figure for simplicity. (B) Hypotheses tested within the SEM model. Two mechanisms were tested, represented by arrows (1) and (2). Arrow (1) was constrained to be negative, representing the potential regulation of pest density through predation (top‐down mechanism). Arrow (2), represented as dashed, was constrained to be positive to evaluate the potential numerical and/or functional responses of predators, so an increase in predation rates to prey density within vineyards (bottom‐up mechanism). In the following analyses, we will focus on the top‐down mechanism with the solid arrow (1). SNH, semi‐natural habitats; MPS, mean patch size; TFI, treatment frequency index.

Following the development of a conceptual model to guide the modeling process, the first step in the SEM process was to relate observed variables to the relevant constructs. The complete SEM represents a specific architecture that includes hypothesized but unmeasured factors, that is, latent variables, and measured variables, that is, observed variables. For instance, landscape diversity was represented by five variables. Among the five variables, the proportion of semi‐natural habitats was associated with the proportion of woody habitats in a 500‐m radius (L1 in Figure 1), the proportion of vineyards was associated with the proportion of organic vineyards in a 500‐m radius (L2), and the mean patch size of vineyards within both 500‐m and 1‐km radii was also grouped (L5). The other two variables represented the proportion of vineyards within a 1‐km radius (L3) and the proportion of semi‐natural habitats within a 1‐km radius (L4), respectively. Local farming practices were categorized by the pesticide use, that is, TFI of synthetic insecticides, fungicides, and herbicides separately, the copper use, and the proportion of ground vegetation cover (Figure 1). Predation types were considered separately depending on the predation type (i.e., egg or pupae or caterpillar predation) and on the sampling period (hereafter P1 and P2 used as suffixes). We distinguished infestation rates depending on the damage type and the sampling period: silk nests corresponded to first‐generation damage, whereas grape perforations corresponded to second‐generation damage of the grape berry moth. All variables were examined for distributional properties and were standardized (i.e., centered‐reduced) as we presumed that each observed variable is linked to one latent variable with standardized Gaussian distributions. Observed variables within a group are all positively intercorrelated (Appendix S1: Figures S4 and S5). A relation model based on a priori knowledge was introduced (see Figure 1A).

We assumed that all the relationships tested were linear. Model estimation was based on maximum likelihood. First, we aimed to determine whether the structure of the measurement model is compatible with the data, independent of the relational model. We used the confirmatory factor analysis (CFA, Rosseel, 2012), where all possible correlations between latent variables are permitted. The goodness of fit of CFA SEM was tested by applying parametric bootstrap on the chi‐square statistics (Laroche, 2022). To further assess model fit, we calculated additional indices, including the root mean square error of approximation (RMSEA) and the comparative fit index (CFI).

The second step consisted of evaluating the relational model by comparison with the CFA SEM. This test evaluates whether the chosen relational model is sufficient to express the correlation structure between latent variables. First, we compared the CFA SEM with a model where correlations between landscape and farming practices were allowed and with direct links from landscape and farming practices to predation types and infestations. This model was accepted. Then, we compared the previous best model with the one without landscape and farming practices correlations; this one was simpler and fit as well the data, so it was kept.

In order to refine the model, we further constrained the relation between predation and infestation in the previous model (Figure 1B). We successively allowed negative and positive relationships between predation and infestation depending on two different hypotheses. The first hypothesis was that predation reduced infestation (H1a), in which case the relationship was negative. The second hypothesis was that pest density drove predation pressure locally (H1b), in which case the relationship was positive. The adequacy of model fit was evaluated using the model chi‐square and its associated *p*‐value, as well as through the examination of beta coefficients constrained within both models (Laroche, 2022).

To account for the spatial autocorrelation observed at local scales (Appendix S2: Table S1), we complemented the SEM analysis with a permutation approach. Plot identities (organic vs. conventional) were randomized 500 times while preserving the dataset structure. For each permutation, 95% and 90% CIs of model coefficients were calculated to assess the robustness of the observed effects. Effects falling outside both the 95% and 90% CIs were classified as robust, while those outside only the 90% CI but within the 95% CI were classified as moderately robust.

Correlations between predation types were extracted from the SEM model, and the significant correlation threshold was calculated based on the number of observations. In our case, the significant correlation value is below −0.4 and above 0.4.

## RESULTS

The predation rate on eggs increased from 19.2% (±20%) (mean ± SD) in early June to 40.4% (±23.9%) in late July. Also, pupae predation rates were higher in summer (51.7% ± 29.6%) than in spring (44.8% ± 23.7%). The average caterpillar predation was 10.2% (±17.5%). The number of silk nests per 100 grape clusters in spring ranged between 0 and 11 and was on average 1.34 (±2.68). In the summer, the number of perforations per 100 grapes ranged between 0 and 45 and was on average 6.26 (±10.10).

The first model, which tested the negative correlation between predation rates and infestation, converged normally (*p*‐value = 0.032), and the constraints were respected. The second model, which tested the positive correlation between predation rates and infestation, converged also normally (*p*‐value = 0.029); however, the constraints were not respected, and the beta coefficients of the effects were set to zero. The first model is therefore the only one whose outputs comply with the constraints. Thus, we report only the results of the first model (see Figure 1).

**FIGURE 2:**
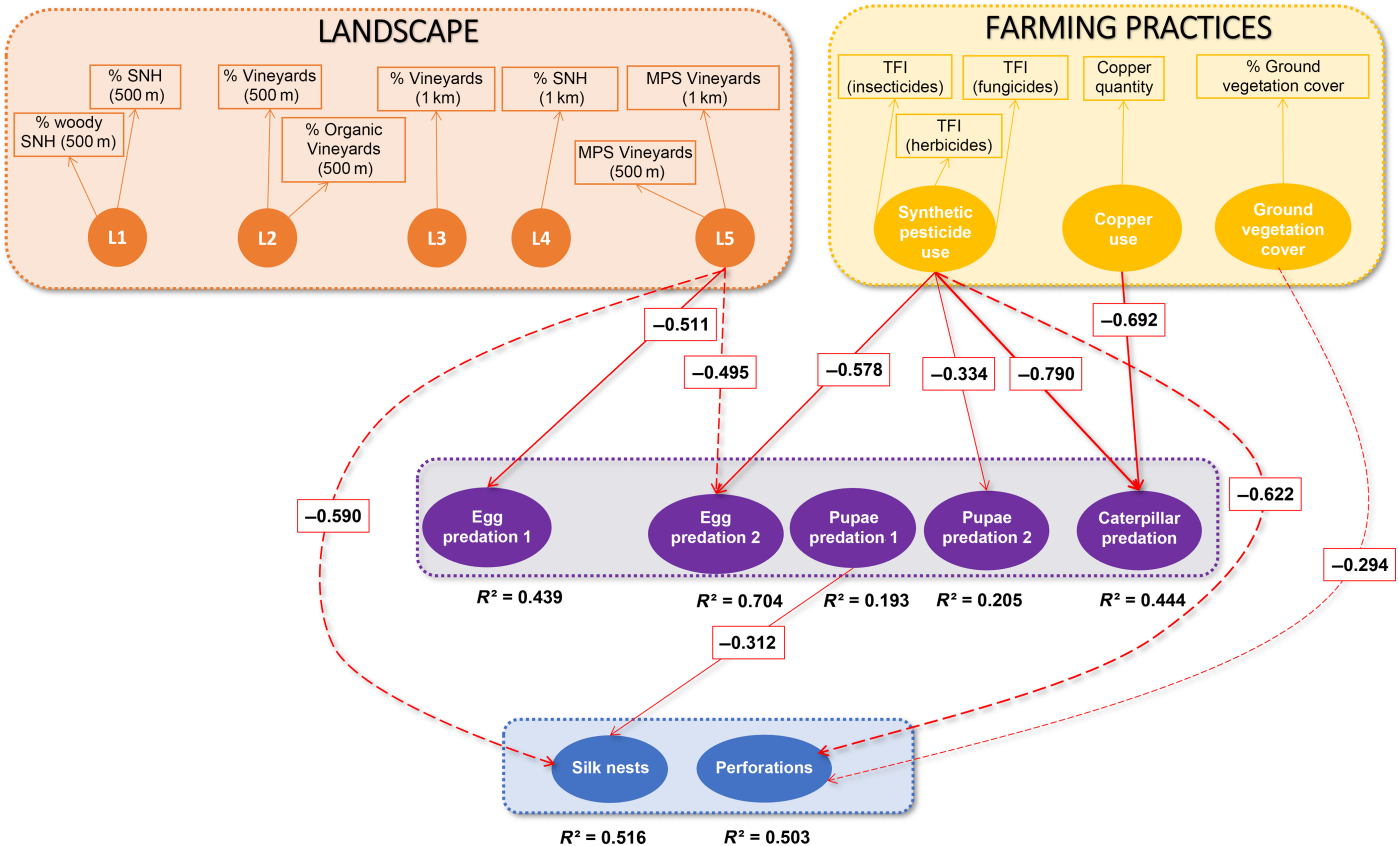
Structural equation model (SEM) showing significant effects and the corresponding standardized estimates. Arrows represent the significant effects identified by the model, with the thickness of the arrow proportional to the magnitude of the standardized estimate. Red arrows indicate negative relationships. Solid lines correspond to effects that are robust after permutation testing (outside the 95% CI), whereas dashed lines represent moderately robust effects (outside the 90% CI). The *R* ^2^ value represents the proportion of variance explained by the model for the endogenous latent variable. Model fit indices for the SEM: RMSEA = 0.08 (*p* = 0.12) and CFI = 0.95. L1: proportion of semi‐natural habitats (500 m) and proportion of woody semi‐natural habitats (500 m); L2: proportion of vineyards (500 m) and proportion of organic vineyards (500 m); L3: proportion of vineyards (1 km); L4: proportion of semi‐natural habitats (1 km); L5: mean patch size of vineyards (500 m and 1 km). SNH, sem‐i‐natural habitats; MPS, mean patch size.

Predation of the different stages of grape berry moth and infestation levels were affected by both landscape and management factors. Caterpillar predation decreased with the increase in copper quantity used and synthetic pesticides (Figure 2). Egg predation (2 periods) was negatively influenced by the increasing mean patch size of vineyards, that is, predation rates were higher in landscapes with smaller vineyards (Figure 2). Egg and pupae predation rates in late July decreased with higher pesticide use. The abundance of silk nests was negatively influenced by the mean patch size of vineyards. The number of perforations decreased with higher pesticide use and the cover of ground vegetation. Finally, only the pupae predation affected negatively the abundance of silk nests (Figure 2).

**FIGURE 3:**
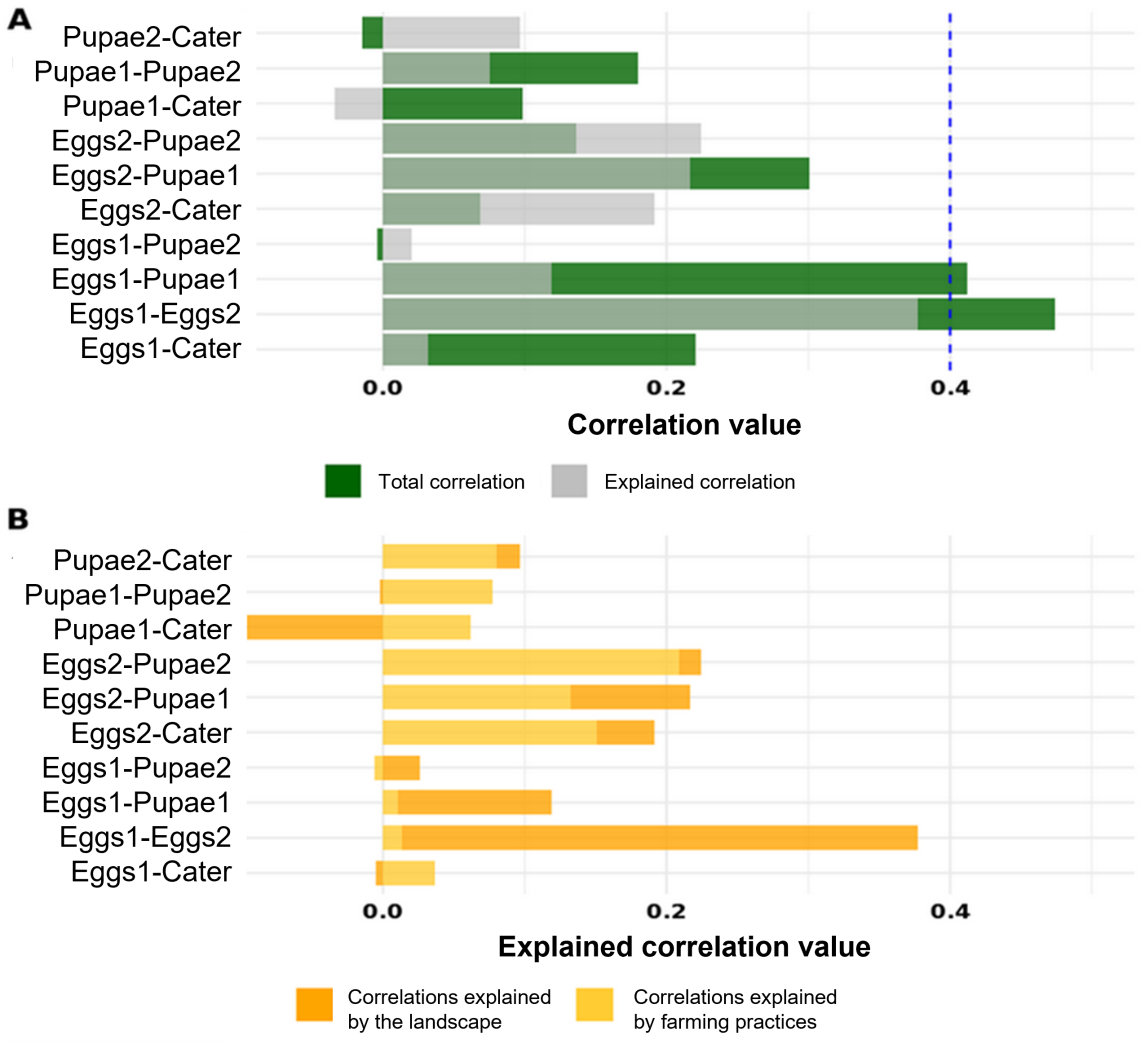
Correlations between the different predation types. Predation types followed by the number 1 correspond to predation rates measured during the first session in early June (spring), while those followed by the number 2 correspond to the predation rates measured during the second session in late July (summer). “Cater” refers to predation on caterpillars using dummy caterpillars, which was measured only in early June. Other predation types were measured in spring. (A) Total and explained correlation values between predation types 2 by 2. The blue dashed line corresponds to the threshold correlation value above which the correlation is considered significant for our dataset. (B) Part of the correlations explained by the landscape and farming practices considered in the model.

Indirect effects of landscape factors or farming practices on pest damage were considered significant only if all the previous effects were significant. However, no significant indirect effects were observed through the SEM. The correlation between the egg predation in early June and late July and the correlation between egg predation and pupae predation were greater than 0.4 and thus considered significant (*r*_Eggs‐Eggs2_ = 0.472 and *r*_Eggs‐Pupae_ = 0.412, respectively, Figure 3). The landscape was the main factor explaining these two correlations (Figure 3).

## DISCUSSION

Contrary to our expectations, grape damage was not reduced by several measures of predation on the different pest developmental stages, suggesting no complementarity over time. Instead, grape moth density and predation rates were directly affected by landscape heterogeneity and farming practices. Predation rates on eggs were negatively affected by increased average size of the vineyards in the landscape, whereas no significant effects of other landscape metrics were sufficiently robust, suggesting a higher contribution of landscape configuration rather than landscape composition to pest suppression. However, almost all predation rates were negatively affected by farming intensity, especially pesticide use which was on average higher in conventional vineyards (higher TFI values). Our results indicate that increasing landscape heterogeneity might favor the natural control of grape berry moth, but only under reduced pesticide use. While synthetic pesticide use is effective in reducing pest damage, it negatively impacts natural enemies' populations, as observed in the reduced predation rates. This creates a dependency on continued pesticide use to control pests, which can compromise long‐term sustainability of pest management strategies. These trade‐offs highlight the need for integrated pest management approaches that reduce pesticide use while promoting biological control through landscape and farming practice diversification.

### The combination of predation types did not lead to higher biological pest control

Our results showed that the abundance of silk nests was limited to some degree by pupae predation, whereas none of the remaining predation types had an effect on pest infestation or crop damage. This indicates that the pupae stage could be the most relevant stage for the biological control of grape moths and should be targeted by conservation biological control strategies. Biological control at the pupae stage has long been considered through parasitic wasps rather than by generalist predators. However, while parasitism has been reported as a significant mechanism of biological control in some studies (Xuéreb & Thiéry, 2006), rates can be highly variable depending on environmental and biological conditions (Pérez Moreno et al., 2000). In this study, we focused solely on predation as a driver of biological control, and we acknowledge the absence of parasitism data as a limitation that could explain part of the variability observed in our model. In addition to predation during the vegetation period, grape berry moth pupae may also be taken by generalist predators from the autumn onward at the time of overwintering (Rusch et al., 2017). However, although immobile and vulnerable prey (Picchi et al., 2017), grape berry moth pupae are protected through anti‐predator mechanisms, such as being deposited in vegetation or buried in the soil (Lindstedt et al., 2019). In our study, the use of sentinel prey, such as exposed pupae, may lead to an overestimation of predation rates compared to natural settings, as prey are more conspicuous and lack protective mechanisms. This limitation is a common concern in predation studies using sentinel prey (Boetzl, Konle, et al., 2020; McHugh et al., 2020). Nonetheless, this approach provides standardized and comparable measures of the potential of pest regulation, which are essential for assessing the relative contribution of predation across different environmental conditions and developmental stages. In addition, our initial hypothesis (H5) predicted that combining predation at different developmental stages of *L. botrana* would result in a higher level of biological pest control, ultimately reducing pest damage. However, this combination implies the involvement of a diverse community of natural enemies, which may also result in intraguild interference, potentially counteracting the expected complementarity (Letourneau et al., 2009; Martin et al., 2013; Tylianakis & Romo, 2010). Then, further analyses considering both predation and parasitism using the detailed community for interaction network approach would be necessary.

One of our main results is that the covariation between different types of predation was positive for all pairs of predation types, except between pupae predation in summer and egg or caterpillar predation in spring. This implies that fields supporting high rates of egg predation are also supporting high pupae predation rates in the spring. Moreover, an increase in egg predation in spring is also related to a higher egg predation in summer. This suggests that egg predation may be consistently carried out across spring and summer, potentially due to a stable taxonomic composition of predator communities, or through changes in community composition, where certain species maintain either a stable *numerical response* (changes in predator abundance with prey density) or a stable *functional response* (consistent predation efficiency at different prey densities) or that environmental conditions that favor pest control are stable across time. Most of the correlations between predation types are explained by landscape metrics in our models, whereas the other non‐significant correlations are explained by farming practices. This result shows the stability of predation potential at certain stages in relation to stable habitats of agricultural landscapes (Perrot et al., 2023). This shows once again the high stakes and potential offered by landscape management (Vialatte et al., 2019, 2021). However, the dominant effects of pest management suggest that landscape management for conservation biological control must be integrated within an overall agroecological approach (Deguine et al., 2023).

### Landscape configuration and pesticide use intensity drive pest damage and density rather than top‐down processes

One of our objectives was to assess the direction of the relationships between predation rates and pest densities. While the negative correlation with pupae predation indicates that predation drives infestation, the absence of more or stronger relationships suggests that pest and predator dynamics are largely decoupled, likely through intensive use of pesticides. This result supports the idea of reducing pesticide use to increase predation potential through beneficial management options for natural enemies.

However, we observed direct effects of landscape configuration metric and farming practices on pest damage and predation rates, suggesting that both factors are crucial to consider for biological pest control.

Our study illustrates that average vineyard size at the landscape scale influences different predation types, that is, egg predation in spring and potentially also in summer, whereas caterpillar and pupae predation were not significantly affected by landscape metrics as expected. These results suggest that different mechanisms are at play, involving different predator communities. For instance, pupae predation is likely driven by carabid beetles (Carabidae), which are active soil‐dwelling predators. Egg predation, on the other hand, involves generalist predators such as earwigs (*Forficula auricularia*, Forficulidae), bush crickets (Orthoptera: Tettigoniidae), and ants (Formicidae), as observed by Reiff et al. (2021), through nocturnal sentinel prey monitoring. Only egg predation increased in landscapes with average smaller vineyards, suggesting active predator communities within fields and around. Several studies showed a biodiversity decrease with increasing mean field size (Clough et al., 2020; Sirami et al., 2019). Indeed, the increase in mean field size leads to a decrease in the density and the diversity of field edges. The nature of these edges can be very different, ranging from vineyard–vineyard edges to permanent semi‐natural vegetation including grassy strips, hedgerows, and forest edges. These edges are less affected by pesticide sprays, less intensively managed, and can therefore be relatively stable habitats for different taxonomic groups and provide additional and/or complementary resources (Dunning et al., 1992), which may encourage predator diversity and movement (Hass et al., 2018). Therefore, smaller fields increase edge density, resulting in a higher potential for biodiversity through supplementation, that is, greater resource availability across habitats; complementation, that is, use of complementary resources for different life stages; and spill‐over processes, that is, movement of species between habitats (Blitzer et al., 2012; Dunning et al., 1992; Tscharntke et al., 2012). Also, field margins have been identified to be important for both common and rare species (Gabriel et al., 2006; Wuczyński et al., 2014) depending on their degree of habitat specialization and mobility. Caterpillar predation is theoretically more related to bird predation (Rusch et al., 2017), which is assumed to be increased with the proximity to semi‐natural habitats and particularly woody semi‐natural habitats (Karp et al., 2018; Maas et al., 2015), facilitating foraging movements from these habitats into neighboring vineyards. In line with previous studies in vineyards, it has been shown that foliage‐gleaning insectivores are more abundant in landscapes with a higher proportion of semi‐natural habitats and that diverse landscapes shelter a higher bird functional evenness and higher potential of avian pest control (Barbaro et al., 2017). However, contrary to our expectations, we did not observe a significant effect of such landscape metrics on caterpillar predation.

According to Rusch et al. (2017), larval nest density, that is, presence–absence of silk nests, was not affected by landscape composition metrics. However, we showed here that it decreased significantly with average vineyard sizes. This result suggests that larger vineyard sizes may dilute pest populations across a wider area, thereby reducing local pest pressure. These findings are in partial agreement with Rosenheim et al. (2022), who reported that increasing field size does not consistently exacerbate pest problems, highlighting the importance of system‐specific mechanisms. One of the main hypotheses explaining such results is that in smaller vineyards, pest populations might experience less dilution due to limited habitat availability, leading to higher observable densities. Additionally, smaller vineyards might offer more favorable microclimatic conditions or reduced predator pressure through competition between natural enemies (Martin et al., 2013), which could further contribute to increased pest densities. Smaller vineyard size may also favor secondary carnivores such as wasps hunting along margins and reducing densities of spiders (Pfister et al., 2015).

Summer damage, that is, the number of perforations in grapes, potentially decreased with a higher proportion of ground vegetation cover within vineyards. This result suggests that at the local level, the ground vegetation cover limits access to the host plant by diluting the host plant or reducing vine vigor and resource quality (Rusch et al., 2017). This local dilution effect may also vary between years according to the mean pest population. Alternative mechanisms, such as reduced pest dispersion or positive effects of ground cover on natural enemies, could also explain these patterns (Blaise et al., 2022; Gardarin et al., 2021).

While our results suggest that both top‐down and bottom‐up processes are at play, we primarily assessed bottom‐up effects on *L. botrana* populations through metrics of damage (silk nests and perforations) as proxies for larval densities, influenced by vegetation diversity and farming practices. However, bottom‐up effects could also indirectly influence generalist predators via the availability of alternative prey. In landscapes with higher vegetation diversity, the abundance of alternative prey might enhance predator populations, potentially increasing pest suppression. These dynamics underscore the need for future studies to explore how prey resource availability shapes predator activity and its cascading effect on pest control (Deere et al., 2024). The prevalence of bottom‐up or top‐down processes may also depend on the year, particularly whether conditions are favorable for pest outbreaks (Almdal & Costamagna, 2023). Our results imply that both top‐down and bottom‐up processes are at play, especially through landscape and farming management. This reaffirms that both play a key role in biological pest control strategies.

One of our main outcomes of the study is that farming practices, especially synthetic pesticide use, directly decreased not only perforations in the summer but also the caterpillar predation rate and predation rates assessed for the second period (eggs and pupae). It is now widely known that pesticide use has negative effects on predator communities and biological pest control (Daelemans et al., 2023; Duflot et al., 2022; Rusch et al., 2010). Our results also show that the use of copper, which is used in both organic and conventional vineyards in our region, had a negative effect on caterpillar predation. We hypothesize that a higher intensity of pesticide use negatively affects functional communities such as generalist predator communities (Rusch et al., 2014) and also potential alternative prey populations, resulting in a disruption of the food web (Chailleux et al., 2014; Koss & Snyder, 2005). Then, while our results demonstrate the potential effectiveness of synthetic pesticides in reducing pest infestation, they also highlight the unintended consequences on functional predator communities. These practices likely disrupt the food web by negatively affecting both natural enemies and alternative prey, as previously suggested. Therefore, reducing dependence on synthetic pesticides is essential for maintaining long‐term ecosystem services, such as natural biological control.

Finally, many studies have now shown that farming practices may counteract landscape effects (Ricci et al., 2019; Tscharntke et al., 2016). In our study, the model with the correlations between landscape metrics and farming practices was not kept, suggesting that these two groups of variables were quite independent. More precisely, the decrease of chemical inputs in organic vineyards is quite heterogeneous and not necessarily less intensive (Etienne et al., 2023). Then, a landscape with a high proportion of organic farming does not imply less‐intensive pest management. The number of tillage operations, historical contaminations (copper contamination in soil for instance), or the number of years since organic conversion are other variables that would be interesting but difficult to characterize at field and landscape levels. Ultimately, our results suggest that intensive farming practices negatively affect both pest moths and their enemies.

## CONCLUSIONS

Nature‐based solutions are increasingly applied in agricultural systems to promote biodiversity and pest control services. Our study underlines the potential of landscape effects to enhance predation across multiple pest developmental stages of *L. botrana*. However, the observed decoupling of predation rates from pest infestation suggests that negative effects of farming practices on predation rates may have outweighed the potential benefits of landscape configurational heterogeneity in promoting effective natural biological control. Predation pressure at the pupae stage appeared to be most relevant for pest suppression, rather than complementarity between predation rates at different life stages. Links between predation rates and pest infestation appear tenuous but point in the direction of predation driving infestation and not as a response to pest abundance.

Further studies should aim to refine the underlying mechanisms through a functional approach by linking the predator and parasitoid communities involved, highlighting their potential complementarity in biological control. This confirms the need for multi‐scale and multi‐taxa functional approaches. Finally, because pest densities and damage do not seem to respond in the same way in spring and summer, it seems necessary to investigate the temporal dynamics of both natural enemies (including predators and parasitoids) and prey in vineyard landscapes.

## CONFLICT OF INTEREST STATEMENT

The authors declare no conflicts of interest.

## Supporting information


Appendix S1:

Appendix S2:

## Data Availability

Data (Tortosa et al., 2025) are available in the Recherche Data Gouv repository at https://doi.org/10.57745/ZA66RI.
