# Supplementary material for: Landscape heterogeneity and pesticide reduction favor predation, but also grape infestation by Lobesia botrana
Source: Ecol Appl. 2025 Jun 3;35(4):e70045. doi: 10.1002/eap.70045 (PMC12130747; doi:10.1002/eap.70045)
Supplement: Supplementary file 1 — Appendix S1: [file EAP-35-e70045-s001.pdf]

ECOLOGICAL APPLICATIONS

Appendix S1

Landscape heterogeneity and pesticide reduction favor predation, but also grape infestation by *Lobesia botrana*

Axelle Tortosa, Aude Vialatte, Fabien Laroche, Adrien Rusch, Martin H. Entling, Brice Giffard

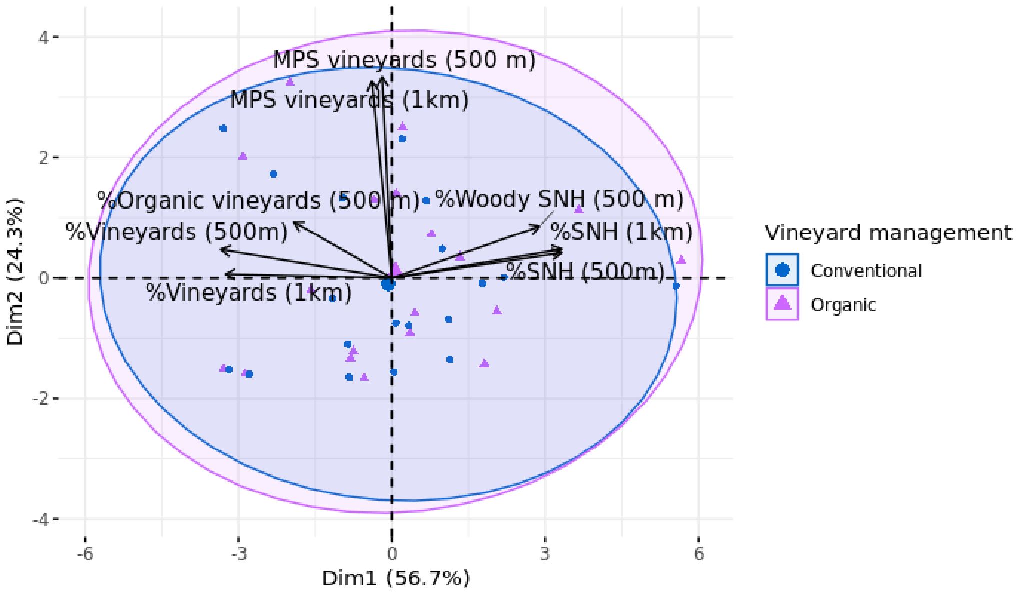

Figure S1: PCA on landscape metrics, showing the overlap of organic and conventional vineyards with respect to the surrounding landscape.

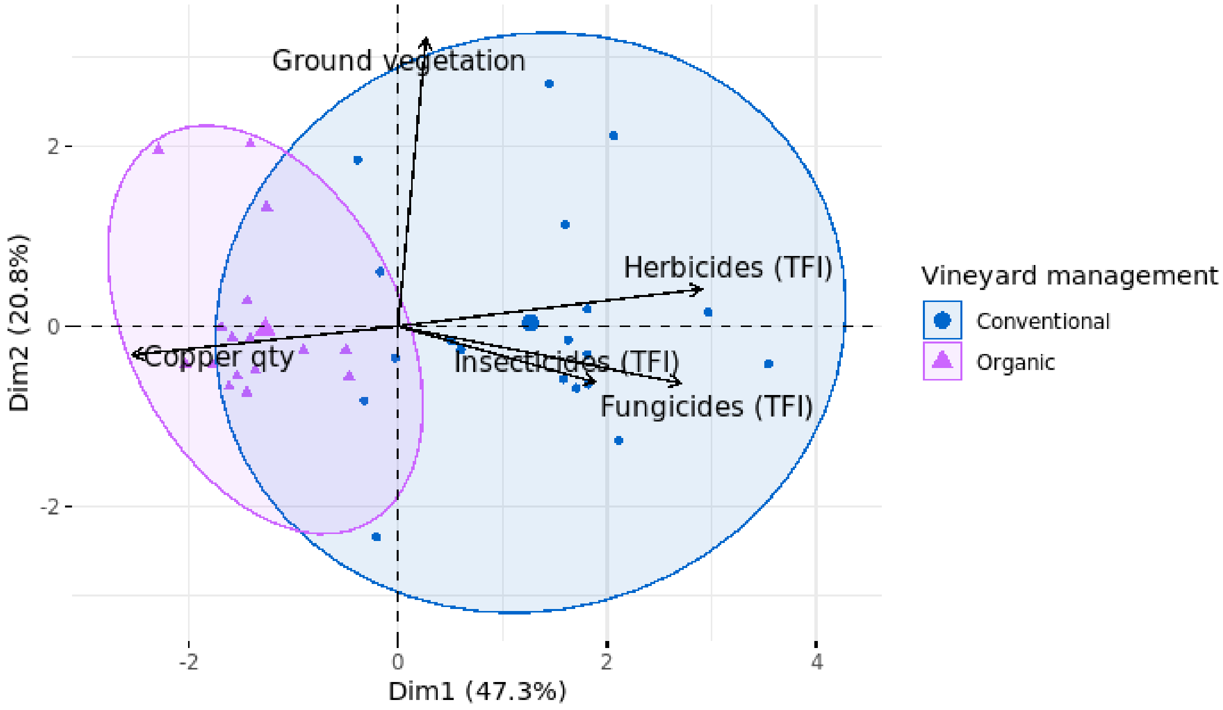

Figure S2: PCA on farming practices, showing the segregation of organic and conventional vineyards with respect to plant protection but not ground vegetation cover.

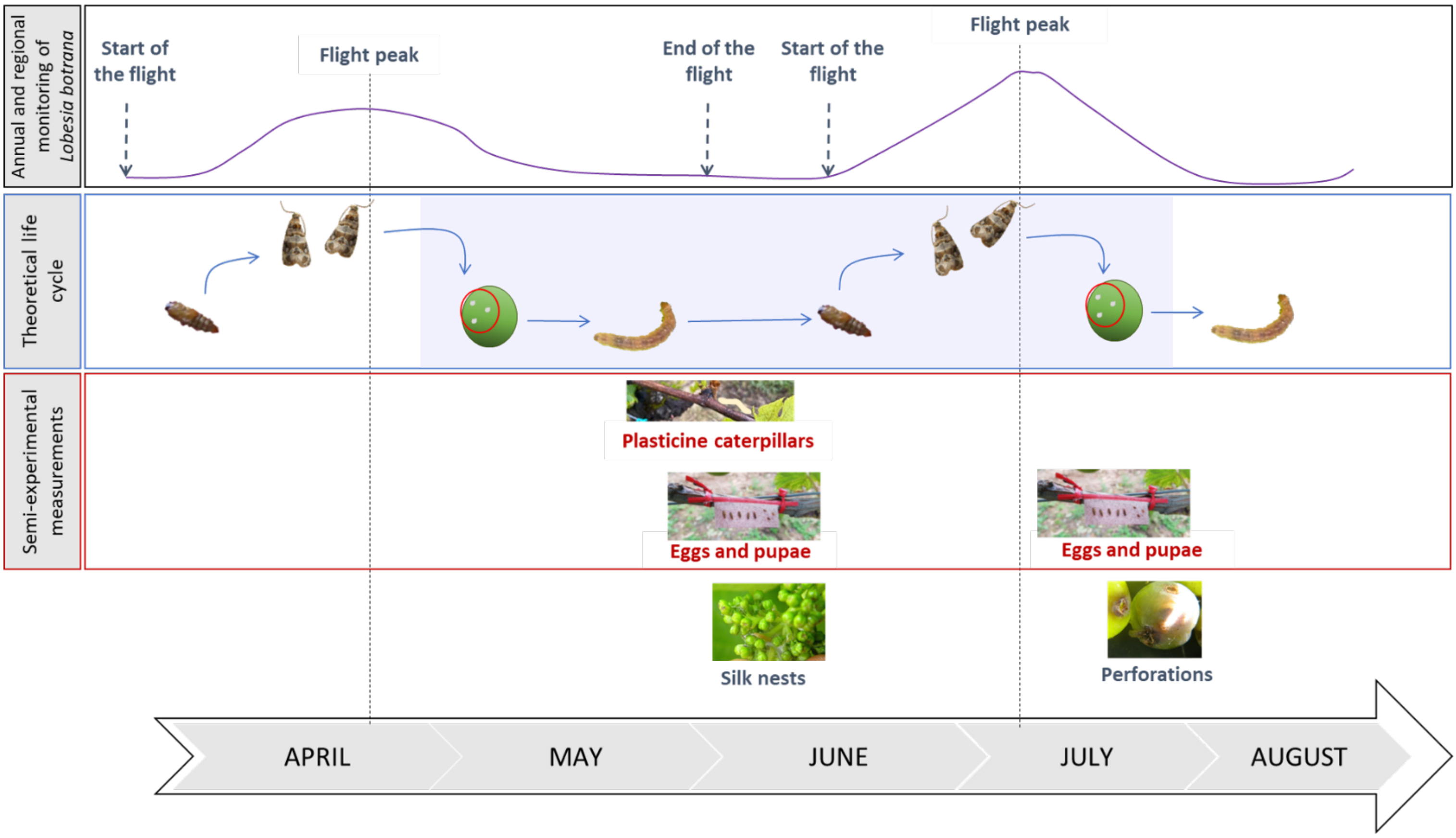

Figure S3: Predation and damage assessment in light of *L. botrana* life cycle and monitoring of dynamics for 2019. In the “Theoretical life cycle” box: photographs of pupae by Adrien Rusch, modified by Axelle Tortosa; photographs of larvae and adult *L. botrana* by Brice Giffard, modified by Axelle Tortosa; photographs of eggs by Axelle Tortosa. In the “Semi experimental measurements” box and below: photograph of plasticine caterpillars by Axelle Tortosa; photographs of eggs and pupae by Adrien Rusch; photographs of silk nests and perforations by Brice Giffard.

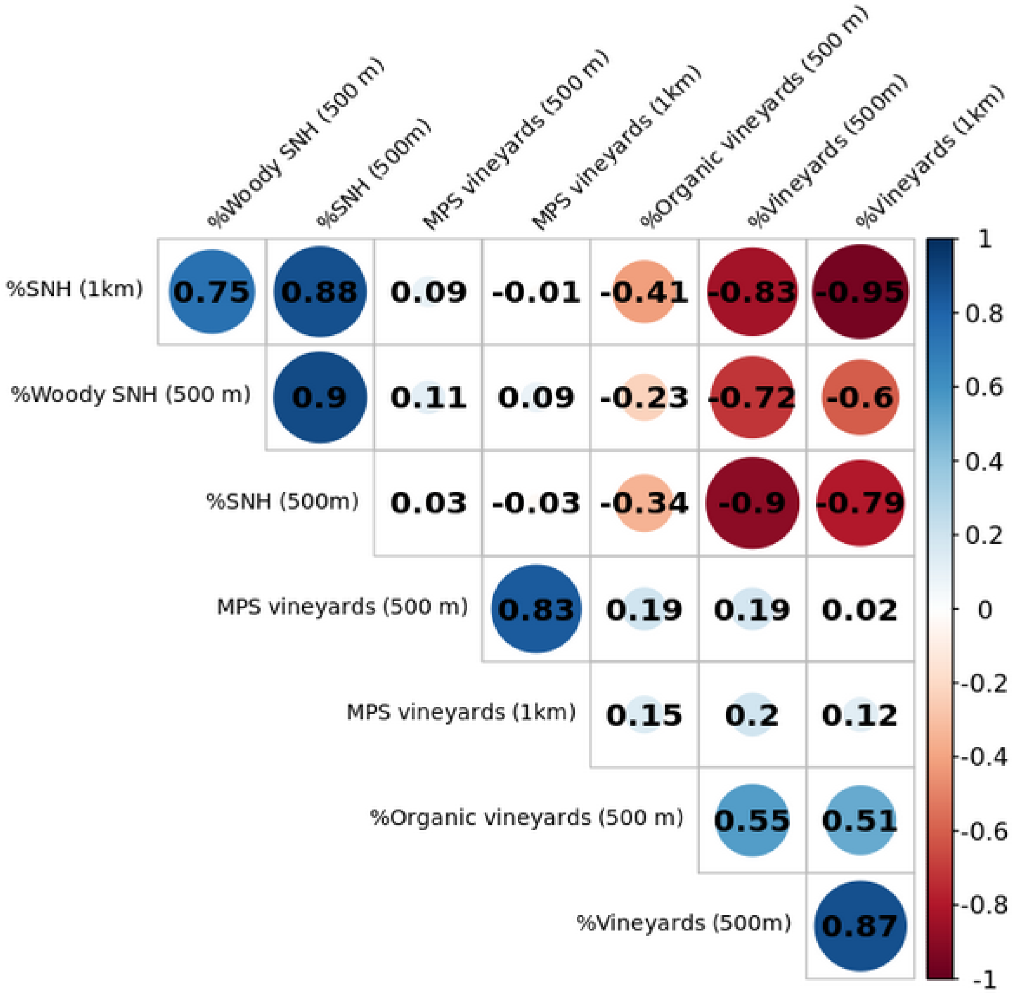

Figure S4: Pearson's correlations between landscape metrics. The mention of 500 m or 1 km corresponds to the buffer radius.

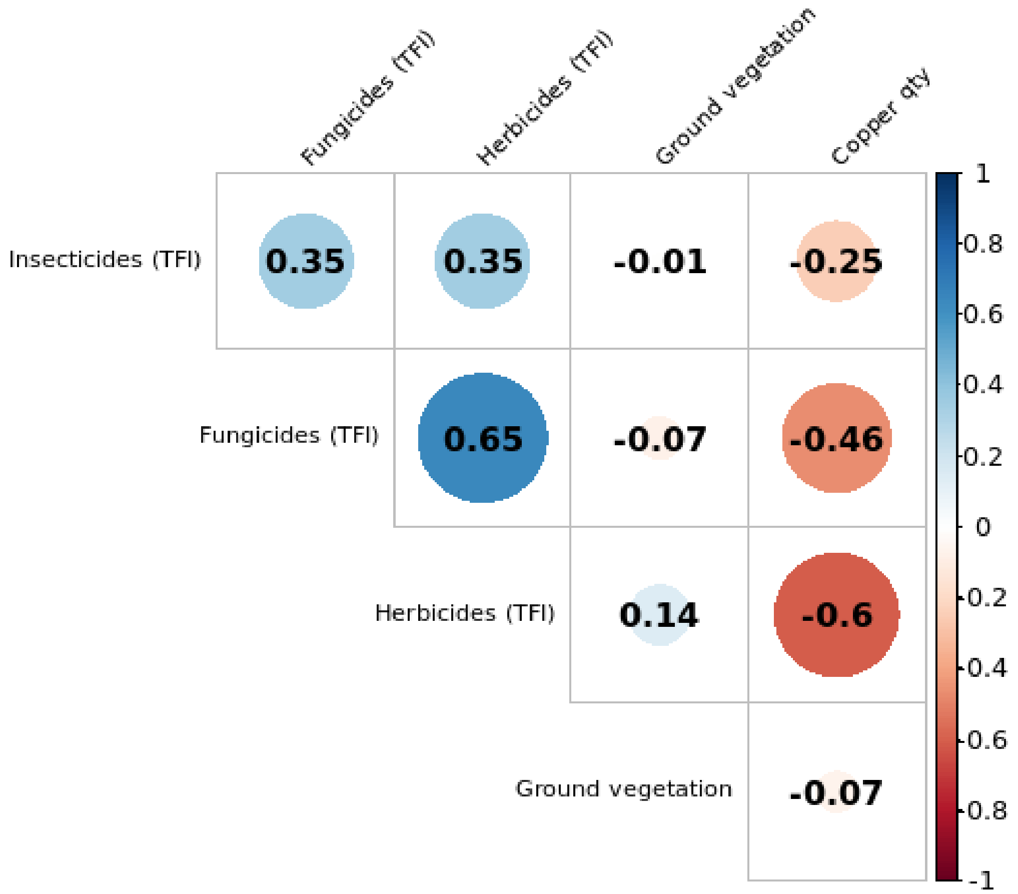

Figure S5: Pearson's correlations between farming practices.
