# Supplementary material for: Landscape heterogeneity and pesticide reduction favor predation, but also grape infestation by Lobesia botrana
Source: Ecol Appl. 2025 Jun 3;35(4):e70045. doi: 10.1002/eap.70045 (PMC12130747; doi:10.1002/eap.70045)
Supplement: Supplementary file 2 — Appendix S2: [file EAP-35-e70045-s002.pdf]

ECOLOGICAL APPLICATIONS

Appendix S2

Landscape heterogeneity and pesticide reduction favor predation, but also grape infestation by *Lobesia botrana*

Axelle Tortosa, Aude Vialatte, Fabien Laroche, Adrien Rusch, Martin H. Entling, Brice Giffard

Spatial autocorrelation analysis

To ensure that the independence assumption was not violated, we evaluated the potential spatial autocorrelation of residuals for all response variables using Moran's I at different distance classes (0–500 m, 500–1000 m, 1000–1500 m, and 1500–2000 m). The results indicated some degree of spatial autocorrelation, particularly within the 0–500 m range, for most variables, except for pupae predation in spring (see Table S1).

Table S1: Spatial autocorrelations of SEM residuals for response variables at different spatial scales. Red p-values show significant spatial autocorrelation.

Response variable	Class	Moran_I	p_value
**Predation Eggs S1**	0-500	0.587	0.004
	500-1000	0.022	0.288
	1000-1500	-0.103	0.586
	1500-2000	0.185	0.145
**Predation Eggs S2**	0-500	0.524	0.008
	500-1000	-0.049	0.354
	1000-1500	-0.109	0.598
	1500-2000	0.054	0.312
**Predation Pupae S1**	0-500	0.205	0.149
	500-1000	-0.072	0.241
	1000-1500	-0.198	0.755
	1500-2000	0.121	0.203
**Predation Pupae S2**	0-500	0.742	0.0003
	500-1000	0.003	0.04
	1000-1500	0.094	0.232
	1500-2000	0.036	0.330

**Predation Caterpillar**	0-500	0.629	0.002
	500-1000	-0.015	0.286
	1000-1500	-0.01	0.415
	1500-2000	0.031	0.345
**Silk nests**	0-500	0.500	0.01
	500-1000	-0.112	0.395
	1000-1500	0.324	0.038
	1500-2000	0.067	0.288
**Perforations**	0-500	0.731	0.0004
	500-1000	-0.117	0.394
	1000-1500	0.122	0.202
	1500-2000	-0.052	0.489

We hypothesize that this spatial autocorrelation is primarily local and arises due to the paired design of the study, where organic and conventional plots were often located near each other (within a 500 m radius, with similar landscapes). Although the SEM framework does not fully account for this design structure and may lead to an overestimation of effects, we addressed this potential limitation using a permutation-based approach.

To mitigate the potential bias introduced by spatial dependencies, we randomized the plot identities (organic vs. conventional) while maintaining the overall dataset structure. For each response variable, we generated 500 randomized datasets and recalculated the SEM models to derive 95% and 90% confidence intervals (CIs) for the coefficients. We chose to present both 95% and 90% CIs (Table S2). Given the small sample size and the spatial structure of our study design, the 90% CI provides a realistic assessment of effect validity.

- Effects that fell outside both CIs (i.e., error > 0.10) were removed from the final SEM.
- For effects validated by the 90% CI but not the 95% CI, we used dashed lines in the SEM figures.
- Effects validated by both the 90% and 95% CIs were represented with solid lines.

This dual representation allows us to convey the robustness of the results while acknowledging the influence of spatial autocorrelation and the paired design.

As shown in Table S2, most coefficients from the final SEM fell outside the CIs derived from the permutation approach, suggesting the robustness of our findings. However, for certain predictors, such as the effect of landscape metrics (proportion of semi-natural habitats, proportion of

vineyards), coefficients did not exceed the CI range. This indicates potential spatial dependencies or design effects.

Overall, this two-step validation process strengthens the reliability of our findings while accounting for potential biases related to the paired design and spatial autocorrelation. Although some effects remain tenuous, the combination of spatial structure and small sample size justifies the use of a 90% CI in addition to the 95% CI.

Table S2: Comparison between estimates from final SEM, CIs 95%, and 90% of estimates based on 500 permutations.

Response variable	Predictor tested	SEM estimate	CI (95%)	CI (90%)	Validation
**Predation Eggs S1**	L5	-0.511	[-0.51, 0.52]	[-0.46, 0.44]	Validated at both
**Predation Eggs S2**	L5	-0.495	[-0.55,0.50]	[-0.46, 0.41]	Validated at 90% only
	Synthetic pesticide use	-0.578	[-0.29, 0.28]	[-0.24, 0.24]	Validated at both
**Predation Pupae S1**	L5	-0.36	[-0.52, 0.49]	[-0.44, 0.45]	Not validated
**Predation Pupae S2**	Synthetic pesticide use	-0.334	[-0.85, -0.68]	[-0.58, -0.56]	Validated at both
**Predation Caterpillar**	L1	0.818	[-0.89, 0.99]	[-1.03, 0.85]	Not validated
	Synthetic pesticide use	-0.790	[-0.66, 0.58]	[-0.76, 0.88]	Validated at both
	Copper use	-0.692	[-0.54, 0.57]	[-0.46, 0.50]	Validated at both
**Silk nests**	L5	-0.590	[-0.63, 0.49]	[-0.57,0.39]	Validated at 90% only
	Pupae predation 1	-0.312	[-0.30, 6.4-10]	[-0.25, 2.4-10]	Validated at both
**Perforations**	L3	1.112	[-1.34, 1.91]	[-1.19, 1.66]	Not validated
	L4	1.666	[-1.93, 2.08]	[-1.55, 1.95]	Not validated
	Synthetic pesticide use	-0.622	[-0.79, 0.96]	[-0.61, 0.14]	Validated at 90% only
	Ground vegetation cover	-0.294	[-0.34, 0.35]	[-0.28, 0.31]	Validated at 90% only
